# Primary health care as a platform for addressing racial discrimination to “leave no one behind” and reduce health inequities

**DOI:** 10.1186/s12939-022-01779-1

**Published:** 2022-11-02

**Authors:** Thomas Hone, Susana Gomez, Mala Rao, Andrêa Ferreira, Shannon Barkley, Theadora Swift Koller

**Affiliations:** 1 Department of Primary care and Public Health, Imperial College London, South Kensington, UK; 2 Department for Gender, Equity and Human Rights, Director General’s Office, World Health Organisation Headquarters, Geneva, Switzerland; 3 Centre for Data and Knowledge Integration for Health (CIDACS), Oswaldo Cruz Foundation, Bahia, Brazil; 4 Primary Healthcare, World Health Organisation Headquarters, Geneva, Switzerland; 5 The Ubuntu Center on Racism, Global Movements, and Population Health Equity, Dornsife School of Public Health, Drexel University, Philadelphia, USA

**Keywords:** Primary Health Care, Health systems, Racism, Discrimination, Health inequities

## Abstract

The health inequities faced by populations experiencing racial discrimination, including indigenous peoples and people of African descent, Roma, and other ethnic minorities, are an issue of global concern. Health systems have an important role to play in tackling these health inequities. Health systems based on comprehensive Primary Health Care (PHC) are best placed to tackle health inequities because PHC encompasses a whole-of-society approach to health. PHC includes actions to address the wider social determinants of health, multisectoral policy and action, intercultural and integrated healthcare services, community empowerment, and a focus on addressing health inequities. PHC can also serve as a platform for introducing specific actions to tackle racial discrimination and can act to drive wider societal change for tackling racial and ethnic health inequities.

## Background

The Constitution of the World Health Organization enshrines “the right to the highest attainable standard of health” [1]. The right to health must be enjoyed by all without discrimination on the grounds of sex, age, race, ethnicity, migration or displacement status, disability, sexual orientation and gender identity or any other status [2]. However, racism, racial discrimination, and social exclusion remain fundamental challenges to improving health and well-being worldwide, addressing health inequities, advancing the right to health, making progress towards the Sustainable Development Goals, and achieving health for all. The health inequities that affect people experiencing racial discrimination, including people of African descent, Roma, and other ethnic minorities, as well as Indigenous Peoples, are unjust, preventable, and remediable [3].

## Main text

Racial and ethnic health inequities are large. An analysis of under-five mortality across 25 low- and middle-income countries between 2010 and 2016 found the median mortality ratio between ethnic groups with the highest and lowest mortality was 3.3 [4]. The COVID-19 pandemic has further exposed racial and ethnic health inequities. Data from the USA and UK in 2020 showed individuals of Black and Asian ethnicity were respectively twice and 50% more likely to infected by COVID-19 than White counterparts [5]. In the USA, age-adjusted data reveals that Hispanic, American Indian, Alaska Native, Native Hawaiian, Other Pacific Islander and Black people are about twice as likely to die from COVID-19 as White Americans [6].

Current day inequities affecting populations experiencing racial discrimination stem from disadvantage accumulated over generations from the legacies of slavery, colonialism, imperialism, ultra-nationalism, ethnic absolutism, and xenophobia. Racism and racial discrimination are fundamental social determinants of health, and they intersect with and compound other determinants [7]. Systemic, or structural racism can arise across society from the interactions of institutions and individuals and can manifest itself in laws, policies and practices, unequal resource distribution and other inequitable practices that limit access to quality health services for some populations [3]. Health systems contribute to structural racism by mirroring the wider structural discrimination present in wider society through geographical, social, cultural differences, and financial barriers to healthcare. Health services can also fail to account for differential patient preferences, or differential treatment of patients, either unintentionally or intentionally, due to unconscious bias or intentional discrimination [8].

Health systems play a fundamental role in safeguarding human rights and alleviating health inequities, and they can serve as platforms for wider societal action to tackle racism and racial discrimination. Health systems orientated towards a comprehensive Primary Health care (PHC) approach, based on the Alma Ata declaration [9], are best placed for this. PHC, as a vision for health systems and society, strives to fulfil the right to health and is indispensable to transforming health systems to tackle racial discrimination and reduce health inequities. This is because PHC is a whole-of-society approach to health that combines multisectoral policy and action, culturally-sensitive approaches to healthcare, the empowerment of people and communities, and integrated healthcare services, with a focus on reducing health inequities and addressing the wider social determinants of health [10]. It is an approach to health that is built on social justice.

In almost all countries, a whole-of-society PHC approach remains a challenge. Comprehensive PHC is often crowded out by more a biomedical and specialist vision of primary care services. Economic and rationalist considerations are often prioritised over social justice. Multi-sectoral action for health and health-in-all policy approaches often receive little attention and can be complex to initiate and sustain. Further action is needed to address the wider social determinants of health. A comprehensive PHC approach based on social justice is essential for addressing racial and ethnic health inequities because it fundamentally aligns with actions to tackle the underlying and root causes of racism, racial discrimination, and health inequities.

There are multiple entry points within a PHC-oriented health system that can specifically tackle racial discrimination. Providing an overview of these opportunities, is the aim of the World Health Organisation’s 2022 research brief (Strengthening primary health care to tackle racial discrimination, promote intercultural services and reduce health inequities) [3]. It is based on a rapid scoping review (only covering published evidence and cases in academic journals, grey literature and policy reports) and aims to contribute to the identification of future research agendas and exploration of entry points for policymakers to tackle racial discrimination.

PHC-oriented health systems have the potential to tackle racism and discrimination through multiple means. For example, the research brief notes the potential for the adoption of specific policy objectives for tackling racial discrimination. It also identifies the opportunities from equity-orientated budgets that invest in disadvantaged areas with greater health needs, participatory budgeting, anti-discriminatory legislation, meaningful participation in decision making, promotion of intercultural and intersectoral approaches, and payment and purchasing systems that incentivize reducing health inequities [11, 12]. There are also examples from actions to increase diversity and representativeness within medical schools, antidiscrimination and cultural competency training for health professionals, and incorporating the perspectives of people experiencing racial discrimination into quality metrics. Community-orientated and controlled primary care services that provide intercultural care are the cornerstone of a strong PHC system that can address racism and discrimination and tackle health equities.

However, the most salient messages relate to the large evidence gaps and challenges that remain. Despite the widespread call for data disaggregation from international human rights mechanisms and civil society, the collection of health data disaggregated by race and ethnicity is inadequate or not available in many countries. This hampers efforts to strengthen health systems by masking inequalities and excluding populations most likely to be marginalized. Developing disaggregated data is essential from a human rights perspective, for meeting the obligations of non-discrimination and equality. It is critical that human rights safeguards are in place for the collection, processing, analysis, and dissemination of data. These safeguards should include ensuring the rights to data protection, including protection of personal identity, registration and self-identification [13]. There also needs to be an increased emphasis on participatory research in health involving persons experiencing racial discrimination. Documentation of health inequities and evaluations of promising interventions are infrequent, but necessary. These are powerful means to put evidence before policymakers.

## Conclusion

The health inequities faced by populations experiencing racial discrimination are significant challenges for countries worldwide. Building strong PHC-oriented health systems, informed by a human rights-based approach, can provide substantial opportunities for embedding actions to tackle racial discrimination, and also for driving wider societal change that can reduce health inequities. These actions complement progress towards SDGs and achieving universal health coverage that all countries have committed to. As part of PHC research, strengthening data collection efforts and research on racial discrimination in health systems and promising interventions will be key to galvanizing action and strengthening PHC in the spirit of leaving no-one behind and achieving health for all.

## Data Availability

Not applicable.

## References

[1] World Health Organization. WHO definition of health, Preamble to the Constitution of the World Health Organization as adopted by the International Health Conference, New York, 19–22 June, 1946. Official Records of the World Health Organization. 1946;(2):100.

[2] Office of the United Nations High Commissioner for Human Rights and World Health Organization. The Right to Health. Fact Sheet No. 31 [Internet]. Geneva, Switzerland: WHO / OHCHR; [cited 2022 Aug 31]. Available from: https://www.ohchr.org/sites/default/files/Documents/Publications/Factsheet31.pdf.

[3] World Health Organization (2022). Strengthening primary health care to address racial discrimination, promote intercultural services and reduce health inequities.

[4] Victora CG, Barros AJD, Blumenberg C, Costa JC, Vidaletti LP, Wehrmeister FC, et al. Association between ethnicity and under-5 mortality: analysis of data from demographic surveys from 36 low-income and middle-income countries. Lancet Glob Health. 2020 Feb 19;8(3):e352–61. 10.1016/S2214-109X(20)30025-5 PMC7034191 32087172

[5] Sze S, Pan D, Nevill CR, Gray LJ, Martin CA, Nazareth J, et al. Ethnicity and clinical outcomes in COVID-19: A systematic review and meta-analysis. EClinicalMedicine. 2020 Dec;29:100630. 10.1016/j.eclinm.2020.100630 PMC7658622 33200120

[6] Hill L, Artiga S. COVID-19 Cases and Deaths by Race/Ethnicity: Current Data and Changes Over Time [Internet]. Kaiser Family Foundation (KFF). 2022 [cited 2022 Sep 23]. Available from: https://www.kff.org/coronavirus-covid-19/issue-brief/covid-19-cases-and-deaths-by-race-ethnicity-current-data-and-changes-over-time/.

[7] Williams DR, Lawrence JA, Davis BA (2019). Racism and Health: Evidence and Needed Research. Annu Rev Public Health.

[8] Institute of Medicine Committee on Understanding and Eliminating Racial and Ethnic Disparities in Health Care. Unequal Treatment: Confronting Racial and Ethnic Disparities in Health Care. In Washington (DC): National Academies Press (US) Copyright 2002 by the National Academy of Sciences. All rights reserved.; 2003. Available from: https://www.ncbi.nlm.nih.gov/books/NBK220358/. 25032386

[9] Hone T, Macinko J, Millett C (2018). Revisiting Alma-Ata: what is the role of primary health care in achieving the Sustainable Development Goals?. The Lancet.

[10] World Health Organization UNC, United Nations Children’s Fund (UNICEF). A vision for primary health care in the 21st century: towards universal health coverage and the Sustainable Development Goals. 2018 [cited 2021 Jun 18]; Available from: https://apps.who.int/iris/handle/10665/328065.

[11] WHO Regional Office for Europe. Toolkit on social participation. Methods and techniques for ensuring the social participation of Roma populations and other social groups in the design, implementation, monitoring and evaluation of policies and programmes to improve their health [Internet]. Copenhagen, Denmark: WHO Regional Office for Europe; 2016 [cited 2022 Feb 5]. Available from: https://www.euro.who.int/en/publications/abstracts/toolkit-on-social-participation.-methods-and-techniques-for-ensuring-the-social-participation-of-roma-populations-and-other-social-groups-in-the-design,-implementation,-monitoring-and-evaluation-of-policies-and-programmes-to-improve-their-health-2016.

[12] Pan American Health Organization. The Knowledge Dialogues Methodology [Internet]. Washington DC: Pan American Health Organization; 2022 [cited 2022 Aug 11]. Available from: 10.37774/9789275124703.

[13] Office of the United Nations High Commissioner for Human Rights. A Human Rights-Based Approach To Data: Leaving No One Behind In The 2030 Agenda For Sustainable Development [Internet]. Geneva, Switzerland: OHCHR; 2018 [cited 2021 Dec 10]. Available from: https://www.ohchr.org/documents/issues/hrindicators/guidancenoteonapproachtodata.pdf.

